# Glycerol Production and Transformation: A Critical Review with Particular Emphasis on Glycerol Reforming Reaction for Producing Hydrogen in Conventional and Membrane Reactors

**DOI:** 10.3390/membranes7020017

**Published:** 2017-03-23

**Authors:** Giuseppe Bagnato, Adolfo Iulianelli, Aimaro Sanna, Angelo Basile

**Affiliations:** 1 School of Engineering & Physical Sciences, Heriot-Watt University, Edinburgh EH14 4AS, UK; gb17@hw.ac.uk (G.B.); a.sanna@hw.ac.uk (A.S.); 2 Institute on Membrane Technology of the Italian National Research Council (ITM-CNR), c/o University of Calabria, via P. Bucci Cubo 17/C, 87036 Rende (CS), Italy

**Keywords:** glycerol production, glycerol steam reforming, conventional reactor, membrane reactor, hydrogen

## Abstract

Glycerol represents an emerging renewable bio-derived feedstock, which could be used as a source for producing hydrogen through steam reforming reaction. In this review, the state-of-the-art about glycerol production processes is reviewed, with particular focus on glycerol reforming reactions and on the main catalysts under development. Furthermore, the use of membrane catalytic reactors instead of conventional reactors for steam reforming is discussed. Finally, the review describes the utilization of the Pd-based membrane reactor technology, pointing out the ability of these alternative fuel processors to simultaneously extract high purity hydrogen and enhance the whole performances of the reaction system in terms of glycerol conversion and hydrogen yield.

## 1. Introduction

**Figure 1:**
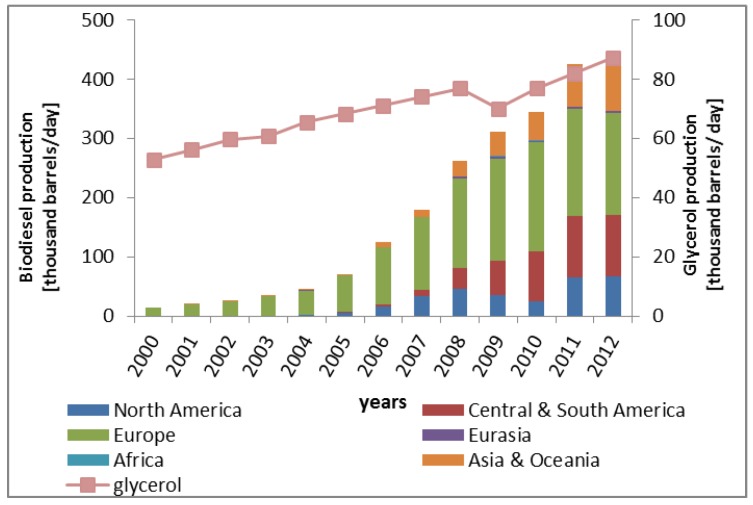
Biodiesel and glycerol production vs. years.

The need for replacing fossil fuels is driving the development of renewable fuels such as biodiesel. In the period 2000–2012, biodiesel production increased from 15 to 430 thousand barrels (Figure 1) [1,2,3]. In the process of producing biodiesel from the transesterification of vegetable oils, glycerol (propane-1,2,3-triol) is produced as by-product. Typically, the production of 100 kg of biodiesel yields approximately 10 kg of glycerol, with purity of around 50%–55% [1].

**Figure 2:**
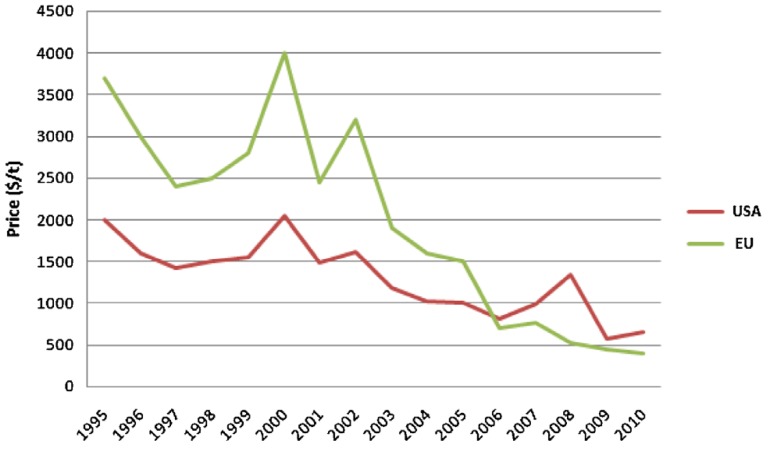
Glycerol price trend in USA and UE. With permission of reprint by Wiley from [1].

The increased production of bio-diesel resulted in a significant fall of glycerol prices from 2000 to 2010 in the European Union (UE) and USA, as can be seen in Figure 2. In particular, the price dramatically decreased from about 3200 $/ton in EU and 2000 $/ton in USA to under 500 $/ton and 600 $/ton, respectively. This was mainly due to a new demand in developing countries as China, India, Russia and Latin American countries, where the glycerol was utilized as raw material in the personal care, pharmaceuticals and food and beverage sectors [4,5,6].

**Figure 3:**
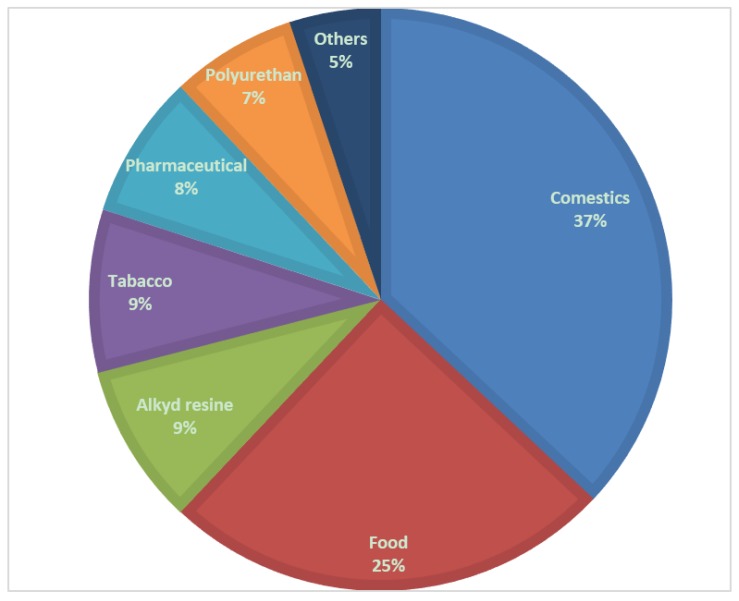
Percentage distribution of the main glycerol applications found in the open literature.

Currently, glycerol is mainly used as an intermediate chemical for the production of a variety of products [7], such as cosmetics, food, pharmaceuticals, etc., as represented in Figure 3.

### 1.1. Glycerol Production

Glycerol can be produced by using different processes and feedstocks. For example, it can be obtained by propylene synthesis via several pathways [8], by hydrolysis of oil or by transesterification of fatty acids/oils. Nevertheless, glycerol production can be also carried out by fermentation with yeast such as saccharomyces cerevisiae, candida, bacteria such as Bacillus subtilis and algae such as dunaliella tertiolecta [9].

#### 1.1.1. Glycerol via Propylene

**Figure 4:**
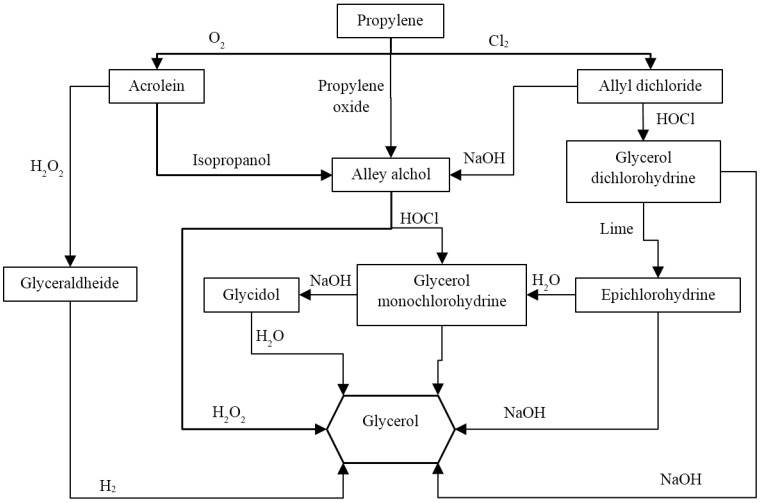
Glycerol production via propylene utilization.

As stated before, several pathways can be used for producing glycerol by propylene [3,10] and Figure 4 sketches two of the principal pathways, which involve the use of O_2_ or Cl_2_.

**Figure 5:**
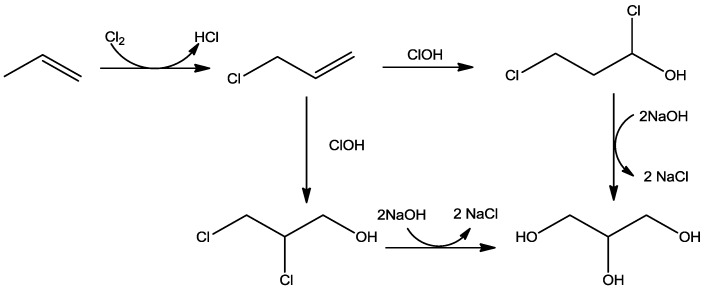
Scheme of the prolylene cholorination process.

In the propylene chlorination (Figure 5), allyl chloride is produced at 510 °C in presence of hypochlorous acid at 38 °C. The allyl chloride reacts to produce glycerine dichlorohydrine. Afterwards, glycerol dichlorohydrine is hydrolysed by caustic soda in a 6% Na_2_CO_3_ solution at 96 °C or directly to glycerine, taking off the epichlorohydrine as an overhead in a stripping column. At the end, the epichlorohydrine is hydrated to glycerine with caustic soda [3] and the process makes possible final glycerol yield of about 90%.

#### 1.1.2. Hydrolysis of Oil

**Figure 6:**
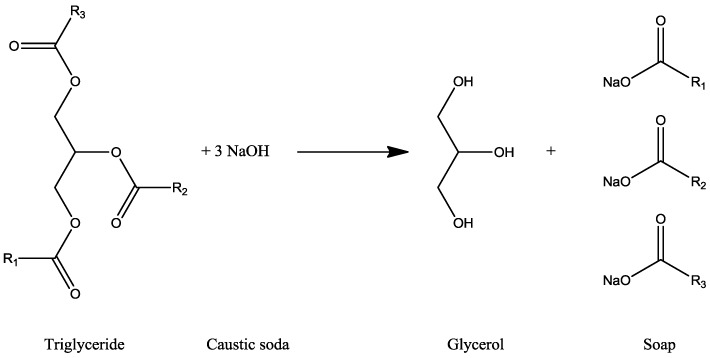
Hydrolysis reaction for glycerol production.

The hydrolysis is a process known since 2800 B.C. and the first industrial plant was built up in 1860 [11]. This reaction takes place between triglyceride and alkaline hydroxide (caustic soda) producing glycerol and soap [12] (Figure 6).

#### 1.1.3. Transesterification of Oil

**Figure 7:**
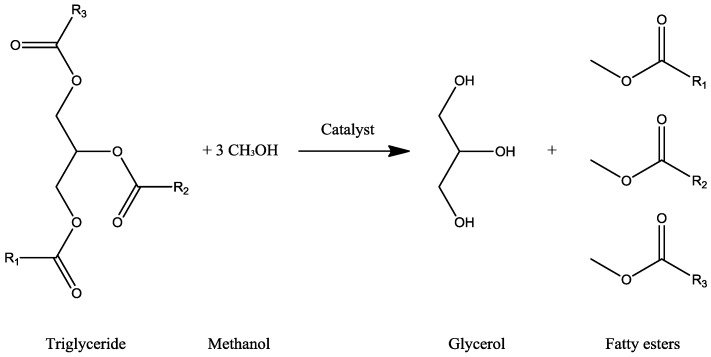
Transesterification reaction for glycerol production.

The transesterification reaction of the beaver oil with ethanol to produce glycerol was conducted in 1864 by Rochieder [5,13]. Figure 7 represents the schematic of the reaction, where methyl-esters from triglyceride (oils) and methanol (alcohol) react to produce glycerol and fatty esters (or biodiesel) [10,14,15].

**Figure 8:**
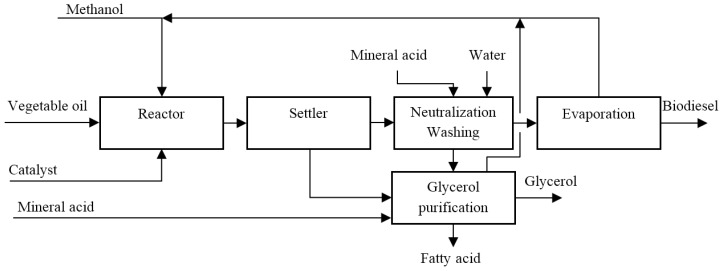
Biodiesel production plant based on homogenous catalyst utilization.

Homogeneous and heterogeneous catalysis can be used to produce bio-diesel and thereby for glycerol production. The process using homogeneous catalysts (in particular, sodium hydroxide or sodium methylate) [13,16] is shown in Figure 8. The first process step involves the reaction between vegetable oils and methanol in presence of the catalyst; subsequently, the glycerol separation from the mixture of products by settler unit takes place. The remnants flow is sent to a unit for the removal of the catalytic component with mineral acids, producing two streams: a glycerol recovery unit and an evaporator, which separates biodiesel from the other products. The glycerol purification unit has three output streams: the first containing 80%–95% of glycerol, the 2nd consisting of water, dissolved salts and unreacted methanol (which is recycled back to the reactor) and the 3rd stream that contains fatty ester [15].

**Figure 9:**
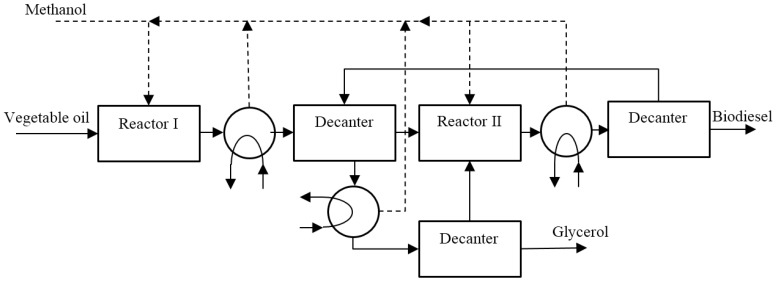
Biodiesel production plant scheme based on heterogeneous catalyst.

The block diagram of triglycerides trans-esterification with heterogeneous catalysts (mainly aluminium and zinc oxides) is reported in Figure 9. To increase the vegetable oil conversion, the process foresees two reaction steps; the first reactor is fed by vegetable oil and methanol. The product stream passes through a heat exchanger to evaporate part of the unreacted methanol, while the remaining stream is sent to a decanter to separate polar (largely glycerol) and non-polar (most vegetable oil and biodiesel) components. In the second reactor, the non-polar stream is reacted for the 2nd time to increase the production of biodiesel and recover the methanol. The product stream passes through the heat exchanger, which removes the entire unreacted methanol, while the decanter separates the biodiesel from polar components. The polar streams of both the first and second polar decanters are sent to another heat exchanger for recovering the remaining methanol present in the mixture, while the residual part is sent into a final decanter for the separation of vegetable oil and unreacted glycerol.

The process of the transesterification reaction through supercritical fluids has been largely studied, even though it is not yet industrialised. One or two reaction steps are possible: in single-step supercritical fluid transesterification, the reaction takes place only after the heating up of reactants to their critical temperature and pressure with triglycerides [17,18]; in two-step subcritical-supercritical fluid transesterification, triglycerides are firstly converted to free fatty acids and by-products, in the hydrolysis reaction. Subsequently, the obtained free fatty acids undergo esterification reaction and produce fatty acid methyl esters in supercritical fluid reaction [19,20].

Depending on the process and feedstock, the glycerol stream is characterized by several compositions; therefore, its characteristics can be identified looking at the different types of feedstocks and reactions utilized during the production process [21,22,23,24,25,26,27,28], as summarized in Table 1.

Tan et al. [25] reviewed most of the advantages and disadvantages of the various techniques of transesterification, which we updated in this work by adding the transesterification reaction whit supercritical fluid (Table 2).

### 1.2. Glycerol Applications

Glycerol can be converted into other compounds with high added value, such as butanol, 1,3-propanediol, 2,3-butanediol, citric acid, lipid, poly(hydroxyalkanoates), acrolein, monoglycerides, etc., via oxidation, reduction, esterification, etc. [27,28,29], by conventional (Table 3) or fermentation process (Table 4).

Therefore, an intensive research work has been addressed in order to investigate the conversion of glycerol to valuable chemicals and Table 3 summarizes some of the most active catalysts for the conversion of glycerol via oxidation, reduction, dehydrogenation, halogenation, esterification and pyrolysis. In detail, the oxidation of glycerol leads to a large number of products such as dihydroxyacetone, glyceric acid, glyceraldehyde, hydroxypyruvic acid, glycolic acid, etc. The control of reaction selectivity represents a key issue to obtain the desired compounds. For example, glyceric acid is an important intermediate for more deeply oxidized products such as tartronic acid and mesoxalic acid [30,34,35,36,37]. The catalytic aerobic oxidation of glycerol has been intensively investigated using monometallic or bimetallic catalysts such as Au, Pt, and Pd in a basic medium and some of the most representative catalysts [30,31,32,33,34,35,36,37] used in this field are reported in Table 3.

Another way for obtaining added value products from glycerol is the reduction reaction. Conventionally, this reaction is carried out at medium/high pressures and temperature ranging from 240 to 270 °C over Cu- and Zn-based catalysts promoted by sulfied Ru catalyst [40]. Furthermore, the reduction of glycerol was also studied over catalysts containing Co, Cu, Mn and Mo as well as over homogeneous catalysts containing W and group VIII transition metals. This reaction has been also studied over Cu-Pt and Cu-Ru bimetallic catalysts at mild conditions under reaction pressures of less than 5 MPa and temperatures of less than 200 °C [45]. It was also reported that the glycerol conversion over Cu-based catalysts was lower than Ru-based catalysts. Indeed, the reduction reaction of glycerol over activated carbon or alumina supported Ru catalysts, combined with various solid acid catalysts such as zeolites, sulfated zirconia, rhenium, niobium and an ion exchange resin, have been recently investigated [42], demonstrating that the combination of Ru-based catalysts and solid acid catalysts exhibit high catalytic activity in high pressure over 8 MPa and between 120 °C and around 200 °C (Table 3).

The production of acrolein from glycerol represents an interesting eco-friendly process, which shows some advantages such as a reduction in the oil exploitation and a low impact towards the environment [46]. Generally, the production of acrolein from glycerol is carried out through acid-catalyzed dehydrogenation over synthetic aluminium phosphate (AlPO_4_) and zeolites with different channel structures (HY and H-ZSM-5) and SiO_2_/Al_2_O_3_ ratio [45,46].

In recent years, a new synthetic route for the preparation of chlorohydrins, by reacting a polyhydroxy aliphatic hydrocarbon with a chlorination agent has been proposed. In particular, Tesser et al. [48] studied homologous chlorinated series of catalysts for glycerol halogenation, such as acetic acid, monochloroacetic, dichloroacetic, trichloroacetic acid, etc. focusing on both activity and selectivity shown by each catalyst.

Table 3 also contains information about one of the most important processes to convert glycerol such as the esterification with acetic acid to produce monoacylglycerol, diacylglycerol and glycerol carbonate. These products are widely utilized in cryogenics, biodegradable polyester and cosmetics [50,51]. Significant acid catalysts can be used for glycerol esterification, including sulfated based superacids, heteropolyacid-based catalysts, tin chloride, zeolite, ZrO_2_ based solid acids, etc. [50,51,52,53,54,55]. Unfortunately, most of them show as main drawbacks the rapid deactivation, complex preparation procedures, low reactivity and expensive costs. As a solution to contrast these disadvantages, graphene and graphene oxide have received great attention because highly active, inexpensive, robust and sustainable solid acid catalyst for glycerol esterification [51].

Last but not least, the pyrolysis of glycerol to produce syngas represents another way to convert the glycerol. In the specialized literature, the pyrolysis of biomass has been widely investigated, but in most of cases only metal-based catalysts have been used. A novel method for syngas production is represented by the microwave-assisted pyrolysis of glycerol over a carbonaceous catalyst, in which the heating method and the operating temperature (between 400 and 900 °C) can influence the catalytic effect of the activated carbons in order to maximize syngas production [55].

Regarding the contents of Table 4, in recent years, the need of developing new and alternative polyol production methods has become of great industrial interest and much attention has been paid to biochemical processes. In particular, Table 4 shows a small overview about the most representative products coming from glycerol conversion by fermentation process.

Among the engineered strains, 1,3-propanediol production from glycerol using *K. pneumoniae* and *E. coli* strains is considered one of the most promising methods [29]. It is influenced by the purity and concentration of the glycerol as well as by fermentation conditions. Furthermore, as reported in Table 4, also 2,3-butanediol can be obtained as a major product of glycerol fermentation by *K. pneumoniae* [65]. Bacteria of the *Enterobacteriaceae* family and the Clostridium genus are useful to convert glycerol to ethanol, even though their yields are relatively low since ethanol represents only a secondary product of the fermentation, while the main products are 1,3-propanediol and 2,3-butanediol. Nevertheless, *E. coli* can transform glycerol to ethanol anaerobically as well as aerobically. Glycerol conversion of about 85% to ethanol (i.e., yield, Table 4) is then possible, demonstrating the potentiality of using *E. coli* as a host for the production of ethanol from glycerol. Butanol represents a key chemical platform, because industrially convertible to acrylates, ethers, and butyl acetate, etc. *C. pasteurianum* can be considered for producing butanol when grown in crude glycerol, although butanol yields and productivity on this substrate is considerably lower than on glycerol. However, another important chemical produced from glycerol fermentation is the dihydroxyacetone, which represents the main active ingredient in all sunless tanning skincare products. As the dihydroxyacetone, glyceric acid is biotechnologically produced mainly by the family of acetic acid bacteria, while recently *E. coli* has been engineered for homofermentative production of lactic acid from glycerol. Succinic acid is widely used for manufacturing health-related products, including pharmaceuticals, antibiotics, amino acids, and vitamins [80]. A recent approach in the production of succinic acid is related to the exploitation of yeast in an aerobic recombinant strain via *Yarrowia lipolytica*, able to produce succinic acid when cultivated on glycerol at low pH. As for the succinic acid, the yeast *Yarrowia lipolytica* has gained much attention in recent years because it is able to metabolize several important industrial and agro-industrial byproducts to produce organic acids such as citric acid, which is considered a weak organic acid, commercially produced by fermentation of molasses.

Oxalic acid is an organic acid useful in industry for the manufacture of paper and detergents [84]. Its production can take place by *Aspergillus niger* growing in crude glycerol waste from biodiesel production plants. *Candida magnoliae* is an excellent mannitol producer using glucose and fructose mixtures as carbon sources. Furthermore, mannitol production from glycerol using *C. magnoliae* can show a consume of 100 g/L of glycerol in 96 h, resulting in 51 g/L of mannitol, corresponding to a yield of 0.51 g/g. Commercial erythritol is produced exclusively via fermentation in substrates containing sugars, such as glucose and fructose, from the hydrolysis of biomass. In case of using residual crude glycerol, an acetate-negative mutant of *Y. lipolytica* (Wratislavia K1) is able to simultaneously produce significant quantities of erythritol and citric acid, while the arabitol production by *D. hansenii* SBP-1 can achieve a yield of 0.5 g/g.

The last part of Table 4 contains indications about the polyhydroxyalkanoates, which have received great attention due to their potential application as renewable, biodegradable, and biocompatible thermoplastics. Poly-3-hydroxybutyrate (PHB) belongs to the group of polyhydroxyalkanoates and represents the most significative example of biodegradable polyesters [92]. Conversion of glycerol to PHBs has reached high production levels due to optimization of strains and fermentations conditions. In particular, fed-batch cultivation improves PHB production by using the *Zobellella denitrificans* MW1, which is characterized by a large amount of PHB from glycerol in presence of NaCl.

However, glycerol can be further used for producing H_2_ via steam reforming, partial oxidation and pyrolysis reactions [93,94,95,96,97,98,99,100,101,102,103,104,105,106,107,108,109,110,111,112,113,114,115,116,117,118,119,120,121,122,123,124,125,126,127,128,129,130,131,132,133,134,135,136,137,138,139,140,141,142,143,144,145,146,147,148,149,150,151,152,153,154,155,156,157,158,159,160,161]. In the next part of the review, particular attention is devoted to this task, with particular emphasis on steam reforming reactions.

## 2. Steam Reforming of Glycerol for Hydrogen Production

### 2.1. Thermodynamic

As also indicated in previous thermodynamic analyses [143,146,161], glycerol steam reforming (GSR) reaction takes place within glycerol and steam to produce hydrogen and carbon dioxide (1):

(1) $$C_{3}H_{8}O_{3}+3H_{2}O\;\Leftrightarrow \;7H_{2}+3{\mathrm{CO}}_{2}\;\Delta {\tilde{H}}_{R}^{0}=129.41\;\mathrm{kJ}/\mathrm{mol}$$

Alongside, secondary reactions such as Water Gas Shift (WGS) (2), methanation (3) and glycerol pyrolysis (4) can occur:

(2) $$\mathrm{CO}+H_{2}O\;\Leftrightarrow {\mathrm{CO}}_{2}+H_{2}\;\Delta {\tilde{H}}_{R}^{0}=-41.40\;\mathrm{kJ}/\mathrm{mol}$$

(3) $$\mathrm{CO}+H_{2}\Leftrightarrow {\;\mathrm{CH}}_{4}+H_{2}O\;\Delta {\tilde{H}}_{R}^{0}=-247.50\;\mathrm{kJ}/\mathrm{mol}$$

(4) $$C_{3}H_{8}O_{3}\;\Leftrightarrow \;4H_{2}+\mathrm{CO}\;\Delta {\tilde{H}}_{R}^{0}=253.50\;\mathrm{kJ}/\mathrm{mol}$$

The GSR reaction (1) evolves towards the products with an increment of moles number and, hence, it is favoured at low pressure and, due to its endothermic nature, it is promoted at higher temperature.

All the aforementioned four reactions are limited by the thermodynamic equilibrium; therefore, the differential equation to the Gibbs free energy for single phase applies:

(5) $$d\left(nG\right)=\left(nV\right)dP-\left(nS\right)dT+\underset{i}{\sum }\mu_{i}dn_{i}$$

In equilibrium closed system, at constant temperature and pressure, Equation (5) can be reduced to (6):

(6) $$\underset{i}{\sum }\mu_{i}dn_{i}=0$$

and if manipulated, it becomes:

(7) $$\ln\underset{i=1}{\prod }{\left(\frac{{\hat{f}}_{i}}{f_{i}^{o}}\right)}^{\nu_{i}}\;=\frac{-\sum_{i}\nu_{i}G_{i}^{o}}{RT}=\ln\left(K\right)$$

**Figure 10:**
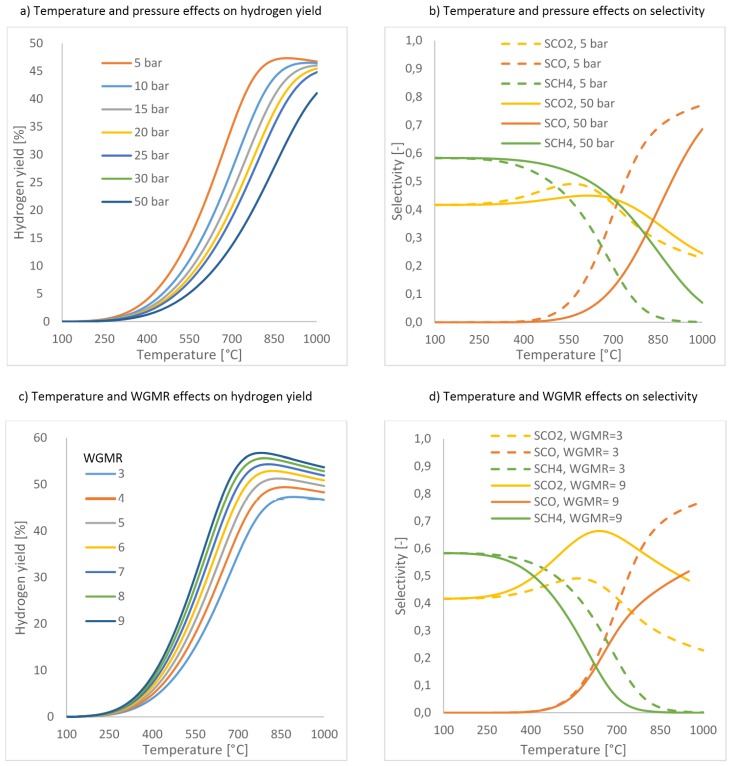
Thermodynamic analysis about GSR reaction performance in terms of hydrogen yield and selectivity: effect of pressure, temperature and water/glycerol molar ratio (WGMR). (**a**) temperature and pressure effects on hydrogen yield; (**b**) temperature and pressure effects on hydrogen selectivity; (**c**) temperature and WGSMR effects on hydrogen yield; (**d**) temperature and WGSMR effects on selectivity.

Equations (6) and (7) can be written for each one of the aforementioned reaction. As a consequence, the thermodynamic data, can be used to show the influence of temperature and pressure on the reaction performance in terms of H_2_ yield and selectivity for *i*-compound, as shown in Figure 10, where H_2_ yield and selectivity are defined as follows:

(8) $$H_{2}\mathrm{yield}=\frac{H_{2}\mathrm{moles}\;\mathrm{produced}}{7\;\mathrm{moles}\;\mathrm{glycerol}\;\mathrm{in}\;\mathrm{feed}\;}\times 100=\left[\%\right]$$

(9) $$S_{i}=\frac{\mathrm{moles}\;\mathrm{of}\;i-\mathrm{compound}}{{\mathrm{CH}}_{4}+\mathrm{CO}+{\mathrm{CO}}_{2}}=\left[-\right]$$

The process endothermicity is clearly depicted in Figure 10a,b, since the best yields were obtained at high temperature. Figure 10b shows that, when the pressure increases the methane production improves (undesired reaction (3)). Furthermore, focusing on carbon dioxide yield at 5 bar, it can be seen that the maximum yield is obtained between 550 and 700 °C, indicating that Equation (2) is favoured among the other reactions. Varying the water/glycerol molar ratio (WGMR) between 3 and 9, the higher the WGMR the higher the hydrogen yield (Figure 10c).

### 2.2. Kinetics and Catalysts Used to Perform GSR Reaction

In the specialized literature about GSR reaction, the heterogeneous catalysts used to perform this process are similar to those used in steam reforming of methane (SRM), such as Ni, Ru, Co, etc. It is worth noting that interesting critical reviews in this field have been already published, highlighting most of the mono and bimetallic catalysts useful for GSR reaction [155,156,157,158].

Table 5 details a brief overview of GSR reaction catalysts, pointing out the values of activation energy, reaction rate and the reaction order for glycerol and water.

A critical issue about GSR reaction is represented by coke deposition and subsequent catalysts deactivation. In the following subparagraph, this task is discussed in brief, but deeper information can be found in Gallo et al. [159], who proposed some catalyst modifications able to reduce catalysts coking, or in Bossola et al. [160], who pointed out a different approach involving a pyrolytic pretreatment step before reformers.

#### 2.2.1. Nichel catalyst in GSR reaction

Ni-based catalysts are studied widely for several reaction processes. Specifically, Cheng et al. [95] proposed a reaction mechanism for the GSR reaction in the presence of Ni on alumina catalyst using kinetics expression of Langmuir-Hinsherwood. The mechanism is described as follows:

$$C_{3}H_{8}O_{3}+X_{1}\Leftrightarrow C_{3}H_{8}O_{3}X_{1}\;H_{2}O+2X_{2}\Leftrightarrow {\mathrm{OHX}}_{2}+{\mathrm{HX}}_{2}\;C_{3}H_{8}O_{3}X_{1}+{\;\mathrm{HX}}_{2}\Rightarrow {\mathrm{CH}}_{2}{\mathrm{OHCHOHX}}_{1}+{\mathrm{CHOHX}}_{2}+H_{2}\;{\mathrm{CHOHX}}_{2}\Rightarrow {\mathrm{COX}}_{2}+H_{2}\;{\mathrm{CH}}_{2}{\mathrm{OHCHOHX}}_{1}+{\;\mathrm{HX}}_{2}\Rightarrow {\mathrm{CH}}_{2}{\mathrm{OHX}}_{1}+{\mathrm{CH}}_{3}{\mathrm{OX}}_{2}\;{\mathrm{CH}}_{2}{\mathrm{OHX}}_{1}+X_{2}\Rightarrow {\mathrm{CH}}_{2}X_{1}+{\mathrm{OHX}}_{2}\;{\mathrm{CH}}_{2}X_{1}+{\mathrm{HX}}_{2}\Rightarrow {\mathrm{CH}}_{3}X_{1}+X_{2}\;{\mathrm{CH}}_{3}X_{1}+{\mathrm{HX}}_{2}\Rightarrow {\mathrm{CH}}_{4}+X_{1}+X_{2}\;{\mathrm{CH}}_{3}{\mathrm{OX}}_{1}+X_{2}\Rightarrow {\mathrm{CH}}_{2}{\mathrm{OX}}_{1}+{\mathrm{HX}}_{2}\;{\mathrm{CH}}_{2}{\mathrm{OX}}_{1}+X_{2}\Rightarrow {\mathrm{HCOX}}_{1}+{\mathrm{HX}}_{2}\;{\mathrm{HCOX}}_{1}+X_{2}\Rightarrow {\mathrm{COX}}_{1}+{\mathrm{HX}}_{2}\;{\mathrm{COX}}_{1}\Leftrightarrow \mathrm{CO}+X_{1}\;{\mathrm{COX}}_{1}+{\mathrm{OHX}}_{2}\Leftrightarrow {\mathrm{CO}}_{2}+{\mathrm{HX}}_{2}+X_{1}\;{\mathrm{HX}}_{2}+{\mathrm{HX}}_{2}\Leftrightarrow H_{2}+2X_{2}$$

Glycerol is absorbed on a catalytic site and it is dissociated into hydrogen and hydroxyl. After that, the absorbed glycerol reacts with the hydrogen absorbed to dissociate into simpler molecules to produce hydrogen and carbon dioxide, which is desorbed at the end of the process. Furthermore, they proposed two kinetic equation models, of which, one of them is based on the reaction mechanism previously illustrated:

(10) $$r=\frac{k_{rxn}P_{G}\sqrt{P_{W}}}{\left(1+K_{G}P_{G}\right)\left(1+\sqrt{K_{W}P_{W}}\right)}$$

where *k_rxn_*, *K_G_* and *K_W_* are equal to 1.33 × 10^−7^ mol·m^−2^s^−1^kPa^−1^, 5.60 × 10^−4^ kPa^−1^ and 0.043 kPa^−1^, respectively.

Wang et al. [14] studied a GSR catalyst based on NiO 24.1 wt %, MgO 26.1 wt % and Al_2_O_3_ 49.8 wt %. At 650 °C, the catalyst exhibits a H_2_ selectivity of 78.5% and glycerol conversion of 88.0%. Dieuzeide et al. [101,102] investigated the influence of Mg presence in Ni-Mg/Al_2_O_3_ catalyst, varying its weight percentage in the range 0%–10%. At 500 °C and WGMR = 3.5/1, they observed the best result in terms of low carbon formation by using the catalyst loaded with 3 wt % of Mg, probably because it favours a better Ni dispersion. Seung-hoon et al. [103] added alkaline metals (K, Ca, Sr) as promoters in Ni/Al_2_O_3_ catalyst. A very low carbon coke formation was obtained by using Sr-Ni/Al_2_O_3_, probably as a consequence of a basicity increment of the whole catalyst. Huang et al. [104] prepared a Ni-based catalysts using commercial Ca-containing Linde-type 5A zeolite (LTA) as support, in presence of Mo–La oxides and CaO to demonstrate that an increase on basic property of Ni/LTA catalysts helps to improving glycerol conversion to syngas and inhibits water–gas shift reaction and methanation during GSR. Gallegos-Suárez et al. [105] tested a NiO (between 5 and 30 wt %) catalysts supported on MgO and CeO_2_ for GSR reaction at different temperature from 250 to 550 °C, weight hourly space velocity (WHSV) = 5.3 h^−1^, WGMR = 16/1, in a fixed-bed reactor. They reached 80% as a maximum glycerol conversion at 550 °C using a 15% NiO catalyst, while glycerol conversion (~15%) decreased due to an increment of carbon coke formation. Shao et al. [106] tested a Ni/CeZr catalyst with different weight percentage of supports to improve the catalytic stability and minimize the carbon coke formation. Kousi et al. [107] investigated the effects of B_2_O_3_ and La_2_O_3_ on Ni/Al_2_O_3_ catalyst during GSR reaction, observing an increment of the hydrogen yield in presence of La_2_O_3_, while by adding B_2_O_3_ the authors noted an inverse result, more pronounced at lower temperatures (~400 °C). Bobadilla et al. [108] studied a Ni-Sn bimetallic catalyst supported over Al_2_O_3_ modified with different promoter (Mg and/or Ce). GSR reaction was then performed at 650 °C, 1 bar and WGMR = 12, observing that the addition of MgO and CeO_2_ made a synergetic effect possible, able to decrease the coke formation by suppression of Lewis acids centres and favouring the WGS reaction. Go et al. [109] studied three different Ni-based catalysts: Ni-Fe-Ce/Al_2_O_3_, Ni-Fe-La/Al_2_O_3_ and Ni-Fe-Cr/Al_2_O_3_. During GSR reaction, Ni-Fe-Ce/Al_2_O_3_ shows low carbon coke formation at high temperature.

Ni and Ni-Pd catalysts supported on Al_2_O_3_-ZrO_2_, Al_2_O_3_-ZrO_2_-La_2_O_3_ and on Olivine have been compared by Yurdakul et al. [110] studying the influence of the support with the temperature. In the temperature range between 600 and 800 °C and at WGMR = 5/1 they reached the maximum H_2_ yield, about 74%, using the Ni-Pd/Al_2_O_3_-ZrO_2_ catalyst. Meanwhile, the presence of La_2_O_3_ decreased the CO_2_ selectivity, resulting unfavourable for the GSR reaction.

#### 2.2.2. Ruthenium Catalyst in GSR Reaction

The reaction mechanism of the GSR reaction in presence of Ru-based catalyst has been proposed by Sundari et al. [111] as follows:

$$C_{3}H_{8}O_{3}+X\;\overset{k_{1};k_{-1}}{\Leftrightarrow }\;C_{3}H_{8}O_{3}X\;C_{3}H_{8}O_{3}X+H_{2}O\;\overset{k_{2}}{\Rightarrow }\;C_{3}H_{8}O_{3}{\mathrm{XH}}_{2}O\;C_{3}H_{8}O_{3}{\mathrm{XH}}_{2}O\overset{k_{3}}{\Rightarrow }\mathrm{Intermediates}\;\overset{k_{4}}{\Rightarrow }\;3{\mathrm{CO}}_{2}+7H_{2}$$

The glycerol (A) is absorbed on the catalyst surface with water (B) creating a complex that, successively, reacts to form CO_2_ and H_2_. In this case, the reaction rate is:

(11) $$r=\frac{k_{1}k_{2}p_{A}p_{B}}{k_{-1}+k_{1}p_{A}+k_{2}p_{B}+k_{1}k_{2}p_{A}p_{B}/k_{3}}$$

At high WGMR, the water partial pressure can be assumed constant (*p_B_* = *p_B_*_0_), so the reaction rate can be rewritten as:

(12) $$r=\frac{k_{R}p_{A}}{1+bp_{A}}$$

where *k_R_* and *b* coefficient can be defined as:

(13) $$k_{R}=\frac{k_{1}k_{2}p_{B0}}{k_{-1}+k_{2}p_{B0}};b=\frac{k_{1}+\left(k_{1}k_{2}p_{B0}/k_{3}\right)}{k_{-1}+k_{2}p_{B0}}$$

As a consequence, for low glycerol partial pressure *bp_A_* << 1, the kinetic rate results to be of the first order (see Equation [13]).

Hirai et al. [112] studied GSR reaction by using Ru catalyst and analysing the effect of different kind of supports (Y_2_O_3_, ZrO_2_, CeO_2_, SiO_2_, MgO and Al_2_O_3_), pointing out that Ru/Y_2_O_3_ made the best performance possible with H_2_ yield about 90% at 600 °C.

#### 2.2.3. Cobalt Catalyst in GSR Reaction

Even though cobalt catalysts do not represent the best solution to carry out GSR reaction, Sanchez et al. [113] studied a bimetallic catalyst, Ni(4 wt %)-Co(4, and 12 wt %), supported on γ-Al_2_O_3_. However, in this study, the authors evidenced that cobalt acts as a precursor, while the catalytic activity is mainly given by Ni. These catalysts favoured the production of H_2_ as the main product, with CO_2_, CO and CH_4_ found in smaller concentrations. In particular, the presence of Co promoted H_2_ production and reduced CO_2_ formation by decreasing the reaction temperature with a depletion of CH_4_ formation. The low Co loading produced the largest H_2_ and CO_2_ amounts at relatively low temperature, with low CO and CH_4_. In contrast, high Co loading maximised the H_2_ production, depleting CO_2_ formation, at relatively low temperature.

Also, Araque et al. [114] used a bimetallic Co-based catalyst (Co-Rh) for the production of hydrogen from GSR reaction. In this case, the cobalt catalyst allowed the selective production of H_2_, where the presence of Rh favoured the stability of the catalyst.

#### 2.2.4. Platinum Catalyst in GSR Reaction

**Figure 11:**
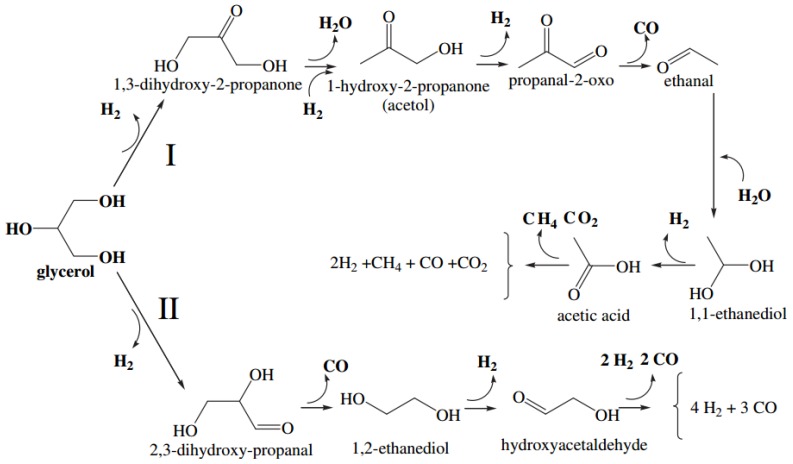
Reaction mechanism during GSR reaction using Pt-based catalysts. With permission of reprint by Elsevier from [115].

Regarding the utilization of Pt-based catalyst in GSR reaction, Pompeo et al. [115] described an interesting mechanism, represented schematically in Figure 11. The mechanism involves two paths: the first pathway (I) consists of a dehydrogenation of glycerol with subsequent dehydration to form acetol; a second dehydrogenation, with a first breaking of C–C bond and subsequent formation of acetic acid (caused by a dehydrogenation and cleavage of the C–C bond). Then, the acetic acid decomposes into CO_2_ and H_2_. The second pathway [II] does not involve dehydration reactions, but mainly cleavage of C–C bonds and dehydrogenations, producing H_2_ and CO. The same mechanism has been proposed for Ni-based catalyst [116].

Pastor-Pérez et al. [117] prepared some bimetallic PtSn/C catalysts whit different Sn/Pt atomic ratios (0.2, 0.3, 0.5, and 0.7) for improving the catalytic activity, selectivity and/or stability. They reached the maximum H_2_ yield (about 36%) for GSR at 1 bar, 350 °C and WGMR = 9/1 and 0.2 as Sn/Pt atomic ratio.

Sad et al. [118] optimized the Pt-based catalyst used for GSR reaction, chaining different type of supports such as SiO_2_, MgO, Al_2_O_3_ and TiO_2_ between 300 and 350 °C. The most important result is the total glycerol conversion, with higher H_2_ yield in case of Pt/SiO_2_ utilization.

#### 2.2.5. Perovskites in GSR Reaction

Surendar et al. [119] doped cobalt based perovskites (LaCo_0.99_X_0.01_O_3_) with X = Au, Ag, Cu and Pt to study GSR reaction between 400 and 700 °C, achieving the best performance in terms of hydrogen yield (~78%) and glycerol conversion (~96%). Furthermore, they demonstrated that the carbon coke formation varies depending on the type of metal dopant as in the following scale: LaCoO_3_ > Au > Ag > Cu ~ Pt.

Ramesh et al. [120] used perovskite catalysts for this reaction at low temperature, specifically LaNi_X_Cu_Y_O_3_ (X between 0.5 and 1 and Y between 0 and 0.5). The goal of the authors was given by the utilization of Ni as reformer catalyst, LaO_3_ to decrease the reaction temperature and Cu to improve the stability of the catalyst and favour a low carbon coke formation. They achieved interesting performance utilizing LaNi_0.9_Cu_0.1_O_3_ at 650 °C in terms of glycerol conversion and H_2_ selectivity (73.0% and 67.3%, respectively).

Mitran et al. [121] used molybdena-alumina based catalysts at 400–500 °C whit WGMR between 9:1 and 20:1 and feed flow rate 0.04–0.08 mL/min. They obtained the best results at maximum percentage of molibdena, WGMR, temperature and lowest feed flow rate, with CH_4_ selectivity less than 5%.

## 3. Innovative Technologies for Producing H_2_ from Steam Reforming of Glycerol

### 3.1. Membranes and Membrane Reactors

Among the various alternative technologies to the conventional systems for producing hydrogen by a green process, membrane reactor (MR) technology plays an important role in terms of Process Intensification Strategy because it involves a unique operation unit to perform both the chemical reaction and the hydrogen separation/purification process [122,123]. Thus, using MRs the plants are more compact with lower investment costs and cost-effective process [124]. The presence of the membrane, in an equilibrium restricted reaction, makes possible to overcome the thermodynamic equilibrium conversion of the equivalent conventional reactor: This is due to the selective removal of a product from the reaction system, acting a shift effect on the reaction itself, which proceeds with a higher products formation (with consequent conversion improvement).

Another functionality of the MRs is represented by the control of reactants addition for permeation through the membrane, avoiding the disadvantage of secondary reactions and increasing the overall reaction.

The membrane can be classified according to its nature, geometry and the type of transport mechanism [125] as follows:

- macroporous membranes, with a pore size greater than 50 nm;
- mesoporous membranes, with a pore size between 2 and 50 nm;
- microporous membranes, with smaller pore size of 2 nm;
- dense membranes, with pore size <0.5 nm.

For dense membranes, the transport mechanism is represented by solution-diffusion, while in porous membranes different types of transport mechanisms often compete with each other to control the process [126,127]. In the following section, we report some of the most common mechanism used for describing a gas permeation process through porous membranes:

- Poiseuille mechanism. It takes place when the average pore diameter is much larger than the mean free path of the molecules; therefore, the collisions within the various molecules are more frequent than those within molecules and porous walls:
  (14) $$J_{i}=-\frac{\varepsilon \times d_{pore}^{2}}{32\times R\times T\times \eta \times \tau }p\nabla p$$
  where *ε* = membrane void fraction, $d_{pore}^{2}$ = pore diameter, *R* = universal constant, *T* = temperature, *p* = pressure, *τ* = tortuosity, ∇*p* = pressure gradient and *η* = viscosity.
- Knudsen diffusion mechanism. When the pores diameters are comparable or less than the mean free path, the quantum momentum is transferred by the collisions between the molecules and the wall of the pores. Applying the kinetic theory of gases to a single straight and cylindrical pore, the Knudsen diffusion coefficient can be defined as:
  (15) $$D_{i,K}=\frac{\varepsilon \times d_{pore}}{3\times \tau }\sqrt{\frac{8\times R\times T}{\pi \times MW_{i}}}$$
  where *ε* = membrane void fraction, $d_{pore}$ = pore diameter, *R* = universal constant, *τ* = tortuosity, *η* = viscosity and *MW* = molecular weight.

As a special field of interest, metallic membranes are particularly involved in hydrogen separation/purification field due to the characteristics of hydrogen perm-selectivity of dense metallic layers [128]. As useful material for membrane fabrication, Pd and its alloys have been extensively studied [129]. However, as high the hydrogen perm-selectivity over all of the other gases as low the permeability (and vice versa), while the cost of the membranes strictly depends on the thickness of membrane material (Pd, Pd-alloy). In the last two decades, composite Pd-based membranes consisting of thin metal films coated over porous supports have been particularly studied because exhibiting high hydrogen permeability and selectivity values depending on the Pd-alloy layer covering the porous support [130,131].

MR technology has been and is particularly used in hydrogen production from the reforming of hydrocarbons and/or alcohols. As a consequence, the utilisation of self-supported and composite Pd-based membranes showing full hydrogen perm-selectivity and high permeability allows for both high-grade hydrogen stream and hydrogen recovery as well.

#### Pd-Based Membrane Reactors for H_2_ Production

**Figure 12:**
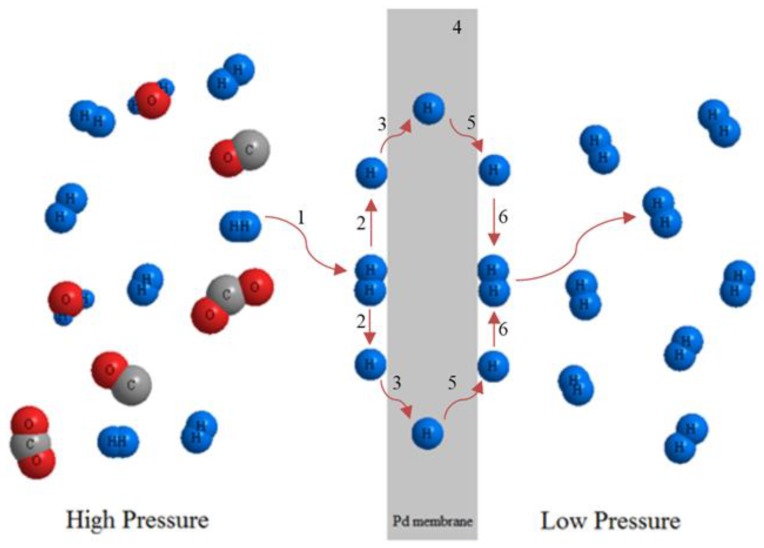
Schematic representation of hydrogen permeation through a dense layer of palladium.

In the last 50 years, several companies as Johnson Mattey moved from the commercialization of unsupported dense Pd-based membranes to composite thin Pd-layer supported on porous substrates, matching the objective of producing more mechanical resistant and cost effective Pd-membranes for potential industrial applications [132]. The transport mechanism of the hydrogen permeation through a dense layer of palladium or its alloy (Figure 12) is represented by the solution-diffusion, which takes place specifically in six steps as resumed below:

1. H_2_ molecules adsorption from the membrane side at higher H_2_ partial pressure;
2. Dissociation of H_2_ molecules on the surface;
3. Reversible dissociative chemisorption of atomic H_2_;
4. Reversible dissolution of atomic H_2_ in the metal lattice of the membrane;
5. Diffusion into the metal of atomic H_2_ proceeds from the side of the membrane at a higher H_2_ pressure to the side at lower pressure;
6. Desorption of re-combined atomic H_2_ into molecular form.

From a theoretical point of view, the solution-diffusion mechanism evolves in three main types of fluxes:

(16) $$J_{1}=k_{1}p_{H_{2},1}-k_{2}{\bar{p}}_{H_{2},1}^{2}$$

(17) $$J_{2}=k_{2}{\bar{p}}_{H_{2},2}-k_{1}p_{H_{2},2}$$

(18) $$J_{3}=D\left(C_{2}-C_{1}\right)$$

Equation (16) represents H_2_ adsorption on the membrane side at higher partial pressure; Equation (17) denotes the dissociation into atomic H_2_, reversible dissociative chemisorption of atomic H_2_ and Equation (18) is the final desorption of recombined H_2_ molecules.

At steady state conditions, the aforementioned fluxes are equal ($J_{H_{2}}$ = $J_{1}$ = $J_{2}$ = $J_{3}$) and, by adding *J*_1_ and *J*_2_:

(19) $$2J_{H_{2}}=k_{1}\left(p_{H_{2},1}-p_{H_{2},2}\right)-k_{2}\left({\bar{p}}_{H_{2},1}^{2}-{\bar{p}}_{H_{2},2}^{2}\right)$$

By taking also into account the average dissolved *H*_2_ concentration, the H_2_ flux can be expressed as:

(20) $$J_{H_{2}}=\frac{1}{\frac{1}{\alpha_{diff}}-\frac{2}{k_{1}}}\;\left(p_{H_{2},1}-p_{H_{2},2}\right)$$

where *α_diff_* is a diffusion coefficient describing the relationship between the resistance of H_2_ transport on the membrane surface and the H_2_ dissociation into the metal lattice,

(21) $$\alpha_{diff}=\frac{Dr^{2}\left(k_{1}/k_{2}\right)}{2\;\delta \;C_{Av}}$$

Consequently, the H_2_ permeating flux can be expressed by the following general equation:

(22) $$J_{H_{2}}=Pe_{H_{2}}\frac{\left(p_{H_{2,ret}}^{n}-p_{H_{2,perm}}^{n}\right)}{\delta }$$

where *Pe*_H__2_ is the H_2_ permeability through the membrane, *p*_H__2*,ret*_ and *p*_H__2*,perm*_ the hydrogen partial pressure in the retentate and permeate sides, respectively, and *n* the exponent expressing the dependence of H_2_ flux to the H_2_ partial pressure (variable between 0.5 and 1) and *δ* the thickness of the palladium layer.

When *n* = 0.5, the transport resistance is represented by the H_2_ dissociation into the Pd-layer, then Equation (22) becomes the Sieverts-Fick law (see Equation (23)):

(23) $$J_{H_{2}}=Pe_{H_{2}}\frac{\left(p_{H_{2},ret}^{0,5}-p_{H_{2},perm}^{0,5}\right)}{\delta }$$

Concerning the temperature influence on the H_2_ permeability, the relationship between the hydrogen permeation rate and the temperature can be described by the Arrhenius law:

(24) $$Pe_{H_{2}}=Pe_{H_{2}}^{o}exp\left(-\frac{E_{a}}{RT}\right)$$

where *Pe^o^*_H__2_ and *E_a_*, are the pre-exponential factor, and the apparent activation energy, respectively.

**Figure 13:**
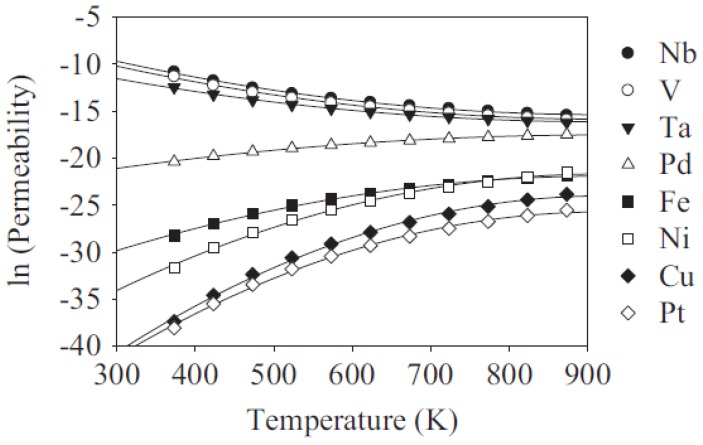
H_2_ permeability through various metals vs. temperature. With permission of reprint by Elsevier from [133].

Steward et al. [133] demonstrated that, when the metals have a body centered cubic (BCC) as a crystal structure (i.e., V, Nb and Ta), they show higher H_2_ permeability than the face centered cubic (FCC) metals such as Pd and Ni. Figure 13 reports the H_2_ permeability through different dense metals versus temperature. As shown in the graph, the H_2_ permeability is inversely proportional to the temperature for V, Nb and Ta, whereas it is directly proportional to the temperature in Pd and Ni.

### 3.2. Glycerol Steam Reforming in Conventional and Membrane Reactors

Most of the literature regarding the glycerol steam reforming reaction for producing hydrogen regards conventional reactors, both in aqueous or gas phase. When performed in gas phase, the process needs atmospheric pressure even though a consistent catalyst deactivation represents the most critical issue. Metals such as Ni and Ru exhibit good catalytic activity but lead to alkanes formation. In contrast, Ir, Co, Cu, Ag, Au and Fe show low catalytic activity. In literature, the catalytic activity scale for gas phase GSR reaction can be represented as in the following: Ru ≈ Rh > Ni > Ir > Co > Pt > Pd > Fe [134]. Among the most active catalysts for this reaction, Rh results to be more effective to steam reforming of hydrocarbons and less susceptible to carbon formation, but Rh-based catalysts are not common in industrial applications owing to their high cost. Zhang et al. [135] performed both steam reforming reaction of ethanol and glycerol over Ir, Co and Ni-based catalysts, determining that Ir-based catalyst is significantly more active and selective toward hydrogen production. Iriondo et al. [136] used alumina-supported Ni-based catalysts, modified with Ce, Mg, Zr and La, pointing out that the differences in catalytic activity are due to the geometric effects caused by the Ni and La or to the close interaction between Ni and Zr. Furthermore, they found that the catalyst deactivation takes place owing to the oxidation of the active catalyst metallic phase. The effect of the supports such as yttria, ceria-zirconia and γ-alumina on catalysts based on Ru and Ru-Me (with Me = Fe, Co, Ni, and Mo) was studied at high temperatures during glycerol steam reforming reaction [137]. It was found that the catalytic properties are notably affected by the nature of the support, resulting in a significant enhancement of H_2_ production turnover rate and product selectivity on the reducible yttria and ceria-zirconia via facilitation of the water-gas shift reaction. The production of pure hydrogen from crude glycerol in a one-stage sorption enhanced steam reforming process (integrating steam reforming of oxygenates and hydrocarbons, WGS and carbonation reactions) was also studied by Fermoso et al. [138]. In a CR packed with a mixture of Ni/Co catalyst derived from hydrotalcite-like material and dolomite as CO_2_ sorbent, they reached an H_2_ yield up to 88% and a hydrogen purity = 99.7 vol % at atmospheric pressure, temperature between 550 and 600 °C and WGMR = 3/1. Other interesting results are reported in a small overview on the most representative literature data about GSR reaction in CRs, in which the performance in terms of glycerol conversion are summarized in function on the reaction temperature and catalyst type used during the reaction (Table 6). As a qualitative comparison, Ru and Ni catalysts seem to have the best catalytic activity towards the reaction, favouring higher glycerol conversion.

As an alternative technology, a few authors investigated GSR reaction in membrane reactors and some results are also reported in Table 6. In particular, the main indication given by this table is that Pd-based MRs can operate at lower temperature than the CRs, meanwhile reaching comparable or better glycerol conversions. This represents an important goal because lower operating temperatures mean higher energy saving and, consequently, cheaper solution for performing the GSR reaction than the conventional processes, with the further advantage of collecting high grade hydrogen. As a special extension of the results related to MRs, Table 7 summarizes other performance in terms of both hydrogen recovery and yield besides other information regarding the operation conditions and the thickness of the dense Pd or Pd-alloy layer.

Lin et al. [148] studied the autothermal glycerol reforming over a Ni/CeO_2_/Al_2_O_3_ catalyst in a MR housing a Pd-Ag/PSS (thickness of the Pd-Ag layer = 33 μm). At 450 °C and WGMR = 5/1, they reached a hydrogen yield of around 35%. In another work, Lin et al. [153] evaluated also the effect of the oxygen addition on the hydrogen yield, reaching a value of around 44%. Chang et al. [153] used a supported porous stainless steel with a Pd-Ag layer deposited via electroless plating (25 μm of dense layer). This membrane was allocated in a MR, which was operated at 450 °C, getting 40% of hydrogen recovery and around 80% of hydrogen yield. Iulianelli et al. [150] allocated a dense and self-supported Pd-Ag membrane (50 μm thick) in a MR module and GSR reaction was carried out over a 0.5 wt % Ru/Al_2_O_3_ catalyst. The experiments were performed at 400 °C, WGMR = 6/1, reaction pressure between 1 and 5 bar and WHSV from 0.1 to 1.0 h^−1^. At 5 bar, around 40% of glycerol conversion was reached with an H_2_ recovery a bit less than 60%. Furthermore, Iulianelli et al. [151] studied the reaction over a Co/Al_2_O_3_ commercial catalyst at 400 °C, WGMR = 6/1, reaction pressure between 1 and 4 bar, producing a maximum glycerol conversion around 94% and an H_2_ recovery a bit higher than 60%.

## 4. Conclusions

Glycerol production can come from different processes and feedstocks, such as by propylene synthesis via several pathways or hydrolisis of fatty acids triglycerides or by transesterification of fatty acids/oils. Furthermore, glycerol can be also produced via fermentation. However, among other renewable and bio-derived sources, glycerol has become an attracting candidate since it constitutes a relevant and alternative solution to produce hydrogen through reforming reactions, performed both in conventional and innovative reactors. In this section, we described the most common processes for obtaining glycerol. Also the role of the catalysts in the reforming reactions of glycerol to produce hydrogen has been considered because demonstrated that the steam reforming performances are much affected by the nature and composition of the catalysts used in the process. Furthermore, as a special case, we illustrated the main benefits of the utilization of an alternative and innovative technology as the membrane reactors in the field of hydrogen production. Indeed, we highlighted that Pd-based MR technology can show superior performance over the conventional reactors, or—in contrast—the same performances but operating at milder conditions, with a consequent advantage in terms of energy saving coupled to the recovery of an high-grade hydrogen stream. In summary, the future perspectives on performing the glycerol reforming in inorganic MRs are listed below:

- The scaling-up of glycerol reforming MRs is one of the most important issues. Developing low-cost, durable and defect-free membranes could represent a viable solution for realistic application of MRs at industrial scale.
- Great attention should be paid to evaluating the effective balance between benefits and drawbacks of applying MR technology to produce hydrogen from glycerol reforming reaction over the conventional processes.
- More wider researches on the lifetime of MRs utilized for carrying out glycerol reforming processes should be undertaken in order to validate them as a potential and alternative solution to the conventional systems at larger scales.

## Figures and Tables

**Table 1:** Characteristic of different glycerol streams depending on initial feedstocks and production reactions (Adapted from [28]).

Feedstock	Glycerol (*w*/*w*)	Methanol (*w*/*w*)	Soap (*w*/*w*)	MONG (*w*/*w*)	Ref.
Waste of palm oil	87.1%	–	–	5.5%	[21]
Jatropha oil	18.0%–22.0%	14.5%	29.0%	11.0%–21.0%	[22]
Soybean oil	63.0%	6.2%	–	–	[23]
Soybean oil	22.9%	10.9%	26.2%	23.5%	[23]
Soybean oil	33.3%	12.6%	26.1%	22.3%	[23]
Waste vegetable oil	27.8%	8.6%	20.5%	38.8%	[23]
Palm oil	80.5%	0.5%	–	<2.0%	[24]
Seed oils	62.5%–76.6%	–	–	–	[25]
Used frying oil	85.3%	–	–	14.7%	[26]

**Table 2:** Advantages and disadvantages of biodiesel and glycerol production by oil transesterification (Adapted from [25]).

Catalysts Group	Type of Catalyst	Advantage	Disadvantage
Homogeneous base catalyst	NaOH KOH	Very fast reaction rate The reaction can occur at mild reaction condition and less energy intensive High conversion can be achieved These catalysts are widely available and economical	The usage limits for oil with less than 0.5 wt % FFA Soap will be formed if the FFA content in the oil is more than 2 wt % Excessive soap formation will reduce the biodiesel yield and cause the problem during the product purification
Heterogeneous base catalyst	CaO MgO	Relatively faster reaction rate than acid catalysed transesterification The reaction can occur at mild reaction condition and less energy intensive Easy separation of catalyst from product High possibility to reuse and regenerate the catalyst	Sensitive to FFA content in the oil due to its basicity property Soap will be formed if the FFA content in the oil is more than 2 wt % Excessive soap formation will decrease the biodiesel yield and cause the problem during product purification Leaching of catalyst active sites may result to product contamination
Homogeneous acid catalyst	H_2_SO_4_ HCl	Insensitive to FFA content and water content in the oil Preferred-method if low-grade oil is used Esterification and transesterification can occur simultaneously The reaction can occur at mild reaction condition and less energy intensive More economical than base catalysed process	Very slow reaction rate Required high reaction temperature and high molar ratio of alcohol to oil Corrosive catalyst such as H_2_SO_4_ used can lead to corrosion on reactor and pipelines Separation of catalyst from product is problematic
Heterogeneous acid catalyst	ZrO_2_ TiO_2_ SnO_2_ Zeolite	Insensitive to FFA content and water content in the oil Preferred-method if low-grade oil is used Esterification and transesterification occur simultaneously Eliminate the washing step of biodiesel Easy separation of catalyst from product High possibility to reuse and regenerate the catalyst Reduce corrosion problem	Complicated catalyst synthesis procedures lead to higher cost Required high reaction temperature, high alcohol to oil molar Ratio and long reaction time are required Energy intensive Leaching of catalyst active sites may result to product contamination
Enzyme	Mucor miehei (Lipozym IM60) C. antarctica (Novozym435) Bacillus subtilis	Insensitive to FFA and water content in the oil Preferred-method flow-grade oil is used Transesterification can be carried out at a low reaction temperature, even lower than homogeneous base catalyst Only simple purification step is required	Very slow reaction rate, even slower than acid catalyzed transesterification High cost Sensitivity to alcohol, typically methanol that can deactivate the enzyme
Supercritical fluid	Noncatalytic	Potential and value of by-products. triacetin and glycerol carbonate were produced in supercritical methyl acetate and dimethyl carbonate technology, respectively High process flexibility of feedstock conditions. Impurities presence of water and FFA do not give any detrimental effects to the product yield	High energy consumption

**Table 3:** Products coming from glycerol conversion by conventional process with related operating conditions.

Reaction Type	Product	Reactant	Catalyst	*p* (bar)	*T* (°C)	Ref.
**Glycerol oxidation**	Dihydroxyacetone	O_2_	Pd–Ag/C	3	80	[30]
		O_2_	Pt/NCNT	–	60	[31]
		O_2_	Au/MWCNT	3	60	[32]
		O_2_	Pt/SiO_2_	1	100	[33]
	Glyceraldehyde	O_2_	Pt/MCN	3	40	[34]
		O_2_	Pt/SiO_2_	1	100	[33]
	Glyceric acid	O_2_	Pt/MCN	3	40	[34]
		O_2_	Pt/SiO_2_	1	100	[33]
		O_2_	AuPdCZ	3	60	[35]
		O_2_	Ag/Al_2_O_3_	5	60	[36]
		O_2_	Au/Al_2_O_3_	5	60	[36]
		O_2_	Pd/Al_2_O_3_	5	60	[36]
		O_2_	Pt/Al_2_O_3_	5	60	[36]
		O_2_	Au/G	5	80	[37]
		O_2_	Au/CNF-R	5	80	[37]
		O_2_	Au/CNS	5	80	[37]
	Glycolic acid	O_2_	Au-Pt	3	60	[38]
		O_2_	AuPdCZ	3	60	[35]
		O_2_	Ag/Al_2_O_3_	5	60	[36]
		O_2_	Au/Al_2_O_3_	5	60	[36]
		O_2_	Pd/Al_2_O_3_	5	60	[36]
		O_2_	Pt/Al_2_O_3_	5	60	[36]
		O_2_	Au/G	5	80	[37]
		O_2_	Au/CNF-R	5	80	[37]
		O_2_	Au/CNS	5	80	[37]
	Hydroxypyruvic acid	O_2_	PtBi/C	–	–	[39]
		O_2_	Au/G	5	80	[37]
		O_2_	Au/CNF-R	5	80	[37]
		O_2_	Au/CNS	5	80	[37]
	Mesoxalic acid	O_2_	PtBi/C	–	–	[39]
		O_2_	Au/G	5	80	[37]
		O_2_	Au/CNF-R	5	80	[37]
		O_2_	Au/CNS	5	80	[37]
	Oxalic acid	O_2_	AuPdCZ	3	60	[35]
	Tartronic acid	O_2_	Ag/Al_2_O_3_	5	60	[36]
		O_2_	Au/Al_2_O_3_	5	60	[36]
		O_2_	Pd/Al_2_O_3_	5	60	[36]
		O_2_	Pt/Al_2_O_3_	5	60	[36]
		O_2_	Au/G	5	80	[37]
		O_2_	Au/CNF-R	5	80	[37]
		O_2_	Au/CNS	5	80	[37]
**Glycerol reduction**	1,2-propanediol	H_2_	Ru/Al_2_O_3_	25	180	[40]
	1,3-propanediol	H_2_	Ru/Al_2_O_3_	80	240	[41]
	Ethylene glycol	H_2_	Ru/Al_2_O_3_	25	200	[42]
		H_2_	Ru/ZrO_2_	80	240	[35]
		H_2_	Ru/ZrO_2_	25	200	[42]
		H_2_	Ru/C	80	130	[43]
		H_2_	3% Ru–0.19% Cu/Al_2_O_3_	100	180	[44]
		H_2_	3% Ru–1% Cu/Al_2_O_3_	80	230	[45]
		H_2_	3% Ru–0.19% Cu/ZrO_2_	100	180	[44]
		H_2_	2.5% Ru–2.5% Cu/Al_2_O_3_	25	200	[42]
**Glycerol dehydrogenation**	Acrolein	–	AlPO_4_-450	1	190–230	[46]
		–	AlPO_4_-650	1	190–230	[46]
		–	H-ZSM-5(50)	1	170–230	[46]
		–	H-ZSM-5(30)	1	170–230	[46]
		–	HY(5.2)	1	170–230	[46]
		–	12 wt % V_2_O_5_, V/P molar ratio of 0.2	1	325	[47]
**Glycerol halogenation**	1,3-dichloropropanol	HCl	Aspartic acid	4.5	100	[48]
		HCl	Glutamic acid	4.5	100	[48]
		HCl	Cystein	4.5	100	[48]
		HCl	Glycolic acid	4.5	100	[48]
		HCl	Diglycolic acid	4.5	100	[48]
		HCl	Thioglycolic acid	4.5	100	[48]
**Glycerol esterification**	Monoglicerides	Acetic acid	Sb_2_O_5_	1	80–120	[49]
	Diacylglicerol	Palmitic acid	ZrSBA-15	1	160–180	[50]
		Acetic acid	Graphene oxide	1	120	[51]
		Acetic acid	ZSM-48	1	120	[51]
		Acetic acid	ZSM-5	1	120	[51]
		Acetic acid	H-mordenite	1	120	[51]
		Acetic acid	WO_3_/ZrO_2_	1	120	[51]
		Acetic acid	MoO_3_/ZrO_2_	1	120	[51]
		Acetic acid	HPW/ZrO_2_	1	120	[51]
		Acetic acid	Cs_2.5_PW	1	120	[51]
	Glycerol carbonate	diethyl carbonate	1-Ethyl-3-methylimidazolium acetate	1	120	[52]
		diethyl carbonate	1,8-diazabicyclo [5.4.0] undecenc-7-ene (DBU)-methanol	1	100	[53]
		diethyl carbonate	CeO_2_	40	90–190	[54]
**Glycerol pyrolysis**	Syngas	–	Bituminous carbon	1	400–900	[55]
		–	Coconut shell	1	400–900	[55]

**Table 4:** Products coming from glycerol conversion by fermentation process with related operating conditions (Adapted from [29]).

Product	Utilization	Organism	Fermentation Mode	Oxygen Availability	Yield (Product/Glycerol)	Productivity	Product Concentration	Ref.
**1,3-Propanediol**	Polytrimethylene terephthalate (PTT), carpets, special textile fibers, monofilaments, films, non-woven fabrics, polybutylene terephthalate (PBT) [56]	C. pasteurianum	Batch	Anaerobic	0.14 g/g	–	4.63 g/L	[57]
		*K. pneumoniae* DSM 2026	Fed-batch	Microaerobic	0.52 mol/mol	1.57 g/L/h	59.50 g/L	[58]
		*K. pneumoniae* LDH 526	Fed-batch	Aerobic	0.52 mol/mol	2.13 g/L/h	102.1 g/L	[59]
		*C. butyricum* F2	Batch	Anaerobic	0.53 g/g	1.05 g/L/h	47.1 g/L	[60]
		*E. coli* K12	Fed-batch	Anaerobic	90.2%	2.61 g/L/h	104.4 g/L	[61]
		*K. pneumoniae*	Fed-batch	Anaerobic	61 mol/mol	2.2 g/L/h	75 g/L	[62]
		*K. pneumoniae* G31	Fed-batch	Microaerobic	0.36 mol/mol	0.18 g/L/h	49.2 g/L	[63]
**2,3-Butanediol**	Plastics, anti-freeze solutions, methyl ethyl ketone production, 1,3-butadiene (used to produce synthetic rubber), diacetyl and to precursors of polyurethane (used in the pharmaceutical and cosmetics industries) [64]	*K. pneumoniae* G31	Fed-batch	Aerobic	0.39 g/g	0.47 g/L/h	70.0 g/L	[65]
**Ethanol**	Food and chemical industries [66]	*C. pasteurianum*	Batch	Anaerobic	0.06 g/g	–	1.87 g/L	[57]
		*E. coli* SY 4	Batch	Microaerobic	85%	0.15 g/L/h	7.8 g/L	[67]
		*C. pasteurianum*	Batch	Anaerobic	0.29 g/g	–	7.85 g/L	[57]
**Butanol**	Paints, lacquers, and resin formulations [68]	*C. pasteurianum*	Batch	Anaerobic	0.36 g/g	–	1.8 g/L	[69]
		*C. pasteurianum* DSM 525	Batch	Anaerobic	0.34 mol/mol	–	7 g/L	[70]
**Dihydroxyacetone**	Skin care products [71]	*G. oxydans* ZJB09112	Fed-batch	Aerobic	88.7%	–	161.9 g/L	[72]
**Glyceric acid**	Chemical and pharmaceutical industries and for the production of polymers [73]	*G. frateurii* NBRC103465	Fed-batch	Aerobic	0.76 g/g	0.81 g/L/h	136.5 g/L	[74]
		*A. tropicalis* NBRC16470	Fed-batch	Aerobic	0.46 g/g	0.71 g/L/h	101.8 g/L	[74]
**Lactic acid**	Food industry, acrylic acid and 1,2 propanediol used in polyester resins and polyurethane [75]	*E. coli* AC-521	Fed-batch	Aerobic	0.9 mol/mol	0.49 g/g/h	85.8 g/L	[76]
		*E. coli* LA02Δdld	Batch	Microaerobic	0.83 g/g	1.25 g/g//h	32 g/L	[77]
**Succinic acid**	Pharmaceuticals, antibiotics, amino acids, vitamins, green solvents, and biodegradable plastics [78]	engineered *E. coli*	Batch	Microaerobic	0.69 g/g	~4 g/g/h	14 g/L	[79]
		*Y. lipolytica* Y-3314	Batch	Oxygen limited	0.45 g/g	–	45 g/L	[80]
**Citric acid**	agro-industrial products [81]	*Y. lipolytica*	Repeated batch	Aerobic	0.77 g/g	0.85 g/L/h	124.2 g/L	[82]
**Oxalic acid**	Manufacture industries, paper and detergents industries [83]	*A. niger*	Batch	Aerobic	0.62 g/g	–	21 g/L	[84]
**Mannitol**	Food and pharmaceutical industries [85]	*C. magnoliae*	Batch	Aerobic	0.51 g/g	0.53 g/L/h	51 g/L	[86]
**Erythritol**	Food industries [87]	*Y. lipolytica* Wratislavia K1	Fed-batch	Aerobic	0.56 g/g	1.0 g/L/h	170 g/L	[88]
**Arabitol**	Food industries [89]	*D. hansenii* SBP1	Batch	Aerobic	0.50 g/g	0.12 g/L/h	14 g/L	[89]
**PHB**	Production of polymers [90]	*E. coli* Arc2	Fed-batch	Microaerobic	–	0.18 g/L/h	10.81 g/L	[91]
		*Z. denitrificans* MW1	Fed-batch	Aerobic	0.25 g/g	1.09 g/L/h	54.3 g/L	[92]

**Table 5:** Typical catalysts for GSR reaction.

Catalyst Type	α *	β **	*E_a_* (kJ/mol)	Ref.
Pt/C	1	–	–	[93]
Co/Al_2_O_3_	0.10	0.4	67.2	[94]
Ni/Al_2_O_3_	0.48	0.34	60.0	[95]
Co-Ni/Al_2_O_3_	0.25	0.36	63.3	[96]
Ni/CeO_2_	0.233	–	103.4	[97]
Ni-ZrO_2_/CeO_2_	0.3	–	43.4	[98]
NiO-MgO/Al_2_O_3_ 45.1-24.1/30.8	0.895	–	131.6	[99]
NiO-MgO/Al_2_O_3_ 34.4-18.5/47.1	0.936	–	74.6	[100]
NiO-MgO/Al_2_O_3_ 24.1-26.1/49.8	0.977	–	37.8	[100]

* reaction order for glycerol, ** reaction order for water.

**Table 6:** Glycerol conversion during GSR reaction in conventional and membrane reactors at various temperatures and catalysts.

Type of Reactor	Catalyst	*T* (°C)	Conversion (%)	Ref.
CR	Ni/Al_2_O_3_	635	100	Demsash & Mohan [139]
CR	Ni/ZrO_2_	600	100	Iriondo et al. [136]
CR	Ni/Al_2_O_3_	920	95	Adikari et al. [141]
CR	Ni/CrO_2_	400	100	Chen et al. [143]
CR	Ni/CeO_2_/Al_2_O_3_	600	92	Buffoni et al. [142]
CR	Rh/CeO_2_/Al_2_O_3_	920	78	Adikari et al. [141]
CR	Pt/CeZrO_2_/Y_2_O_3_	600	81	Cui et al. [143]
CR	Co/Al_2_O_3_	550	65	Cheng et al. [94]
CR	Rh/Al_2_O_3_	630	85	Chiodo et al. [146]
CR	Ru/Al_2_O_3_	720	100	Byrd et al. [147]
CR	Ni/CeO_2_/Al_2_O_3_	800	96	Lin et al. [148]
CR	Ni/Cu/Al	650	91	Wang et al. [149]
CR	Co/Al_2_O_3_	400	40	Iulianelli et al. [150]
CR	Ru/Al_2_O_3_	400	45	Iulianelli et al. [151]
Pd-Ag/PSS MR	Ni/CeO_2_/Al_2_O_3_	450	27	Lin et al. [148]
Pd-Ag MR	Co/Al_2_O_3_	400	94	Iulianelli et al. [150]
Pd-Ag MR	Ru/Al_2_O_3_	400	57	Iulianelli et al. [151]
Pd-Ag/PSS MR	Ni/CeO_2_/Al_2_O_3_	400	24	Chang et al. [153]
Pd-Ag/PSS MR	Ni/CeO_2_/Al_2_O_3_	450	95	Lin et al. [154]

**Table 7:** Experimental data from literature about glycerol reforming in membrane reactors.

Type of Reactor	Pd or Pd-Alloy Layer	*T* (°C)	*p* (bar)	H_2_ Recovery ^(a)^	H_2_ Yield ^(b)^	Ref.
Pd-Ag/PSS	33	450	–	–	35	Lin et al. [148]
Pd-Ag MR	50	400	1.0	63	39	Iulianelli et al. [150]
Pd-Ag MR	50	400	5.0	56	28	Iulianelli et al. [151]
Pd-Ag/PSS	25	450	3	40	80	Chang et al. [153]
Pd-Ag/PSS	27	4	1	–	44	Lin et al. [154]

^(a)^ H_2_ Recovery = Molar ratio between the CO*_x_*-free hydrogen permeated stream and the total hydrogen really produced. ^(b)^ H_2_ Yield = Molar ratio between the hydrogen stream in the permeate side and the total hydrogen theoretically producible from the stoichiometry of reaction.

## List of Acronyms and Symbol

BCC	Body centered cubic
$d_{pore}$	Pore diameter
*E_a_*	Activation energy
$f_{i}^{o}$	Fugacity for *i*-component
FCC	Face centered cubic
FFA	Free fatty acid
G	Free Gibbs energy
GOSR	Glycerol oxidative steam reforming
GSR	Glycerol steam reforming
$J_{H_{2}}$	Hydrogen flux through the membrane
*K*	Equilibrium constant
*k_i_*	Kinetic constant for *i*-reaction
LTA	Linde-type 5A zeolite
MONG	Matter organic non-glycerol
MR	Membrane reactor
MW	Molecular weight
OGMR	Oxygen glycerol molar ratio
P	Pressure
*p*_H2*,ret*_	Hydrogen partial pressure in the retentate side
*p*_H2*,perm*_	Hydrogen partial pressure in the permeate side
$Pe_{H_{2}}$	Hydrogen permeability through the membrane
$Pe_{H_{2}}^{o}$	Pre-exponential factor
R	Universal ideal gas constant
S	Entropy
SRM	Steam reforming of methane
T	Temperature
TR	Traditional reactor
WGS	Water gas shift
WGMR	Water glycerol molar ratio
WHSV	Weight hourly space velocity
*δ*	Palladium thickness
*ε*	Membrane void fraction
*τ*	Tortuosity
*∇p*	Pressure gradient
*η*	Viscosity
$\nu_{i}$	Stoichiometric coefficient for *i*-component
